# Evolutionary morphology of the rattlesnake style

**DOI:** 10.1186/1471-2148-9-35

**Published:** 2009-02-10

**Authors:** Jesse M Meik, André Pires-daSilva

**Affiliations:** 1 Department of Biology, The University of Texas at Arlington, Arlington, TX, USA

## Abstract

**Background:**

The rattlesnake rattling system is an evolutionary novelty that includes anatomical, behavioral, and physiological modifications of the generalized pitviper tail. One such modification, the formation of a bony clublike style at the terminal region of the caudal vertebrae, has not previously been examined in a phylogenetic context. Here we used skeletal material, cleared and stained preparations, and radiographs of whole preserved specimens to examine interspecific variation in style morphology among 34 rattlesnake species.

**Results:**

Evolutionary Principal Components Analysis revealed an inverse relationship between caudal segmental counts and style size, supporting the hypothesis that bone from caudal vertebral elements was reallocated to style formation during the evolution of this structure. Most of the basal rattlesnake species have small styles consisting of few compacted vertebral elements; however, early in the rattlesnake radiation there appears to have been two independent transitions to relatively large, pronged styles consisting of multiple coalesced vertebrae (once in *Sistrurus catenatus*, and once in *Crotalus *following the divergence of the Mexican long-tailed rattlesnakes). In terms of style shape, the two most divergent species, *C. catalinensis *and *C. ericsmithi*, provide insight into the possible relationship between style and rattle matrix morphology and lineage-specific evolutionary strategies for retaining rattle segments.

**Conclusion:**

The considerable interspecific variation in rattle morphology appears to correspond to variation in the bony style. We hypothesize that style morphology evolves indirectly as an integrated module responding to adaptive evolution on matrix morphology.

## Background

The rattlesnake rattle is a complex aposematic sound-producing structure, composed of keratinous, multilobed, interlocking segments that is presumed to have evolved once [1,2] and as an integrated anatomical, physiological, and behavioral system is a key innovation uniting a diverse radiation of venomous New World pitvipers (genera *Crotalus *and *Sistrurus*). Aside from early embryonic and ontogenetic descriptions [e.g., [3-6]] very little is known of the development of the rattle system. Most recent studies have focused on physiological and auditory elements of the rattle [e.g., [7-9]]; the genetic basis of morphological development remains unknown. The site of actual rattle segment formation is the end-body, the living basal rattle segment that includes the style, the matrix (i.e., fibrous connective tissue that forms the rattle segment mold), and epithelial tissues. Although the term 'matrix' has often been used in reference to the entire structure that secretes the rattle producing keratins [10], we herein use 'matrix' in reference to the soft tissues of this structure, and 'style' in reference to the fused caudal vertebrae embedded within the matrix. Rattle segment formation is tied to pulses of matrix tissue growth and resorption during the ecdysis cycle, and ontogenetic changes in size and morphology of the matrix influence rattle morphology. Considerable interspecific variation exists in the overall size and form of the rattle; some rattlesnake species have tiny, scarcely audible rattles, while others have proportionately large, loud rattles [10,11]. This variation likely involves other system components as well and these integrated elements may collectively provide an ideal opportunity to study the evolution of a structural novelty in a vertebrate taxon.

Here we evaluate morphology and evolution of an anatomical component of the rattlesnake rattle system, the bony clublike style, or shaker, at the terminus of the caudal vertebrae. The rattle style is comprised of a combination of fused and modified caudal vertebrae and further exostosis of bone [6,10]. It provides a point of insertion for the powerful tailshaker muscles [6], and presumably provides stability for the motion-dynamic rattle [12], as well as potentially influences the morphology of the end-body. In general structure, the style of an adult rattlesnake is vaguely arrowhead-shaped, with proximal notches, and two distally projecting prongs that extend into the distal lobe of the matrix. Observations of style development suggest that the style includes the coalescence of 8–12 of the terminal vertebrae [6]; however, the precise number is difficult to determine due to the fusion of vertebrae and accretion of extravertebral osseous tissue. Studies of matrix morphology in insular populations of rattlesnakes that have undergone vestigilization of the rattle suggest that morphology of the grooves and lobes of the external matrix surface is vital for the retention of interlocking, sound-producing segments in adult rattlesnakes [13]; thus the relationship between matrix and style morphology may provide insight into the evolution and morphological integration of rattle system components.

Zimmerman and Pope [6] documented differences in timing and degree of development of the style between *Crotalus atrox*, *C. adamanteus*, and *Sistrurus catenatus*, providing evidence that heterochronic changes in strength and modulation of genetic pathways involved in style formation may be linked to evolutionary changes in the rattlesnake rattle. Furthermore, style formation appears to occur late in prenatal development, as well as postnatally, and the structure likely continues to accrue osseous tissue into adulthood [6]. Klauber [10], in reference to interspecific differences previously noted by Zimmerman and Pope [6], suggested that the style might be a potentially informative character in deciphering rattlesnake relationships. Although he did not present detailed data, Brattstrom [14], in his landmark study of pitviper osteology, did not find consistent differences or similarities among species of rattlesnakes. Recently, Savitzky and Moon [12] examined vertebral variation along the length of the rattlesnake tail in *C. atrox *and related it to functioning of the various shaker muscles in maintaining structural stability during high frequency contractions. The gross morphology of the rattlesnake style has been described elsewhere [e.g., [4,6]]; our purpose is to explore general transformations and interspecific variation in morphology of the style in a phylogenetic context.

## Methods

**Figure 1:**
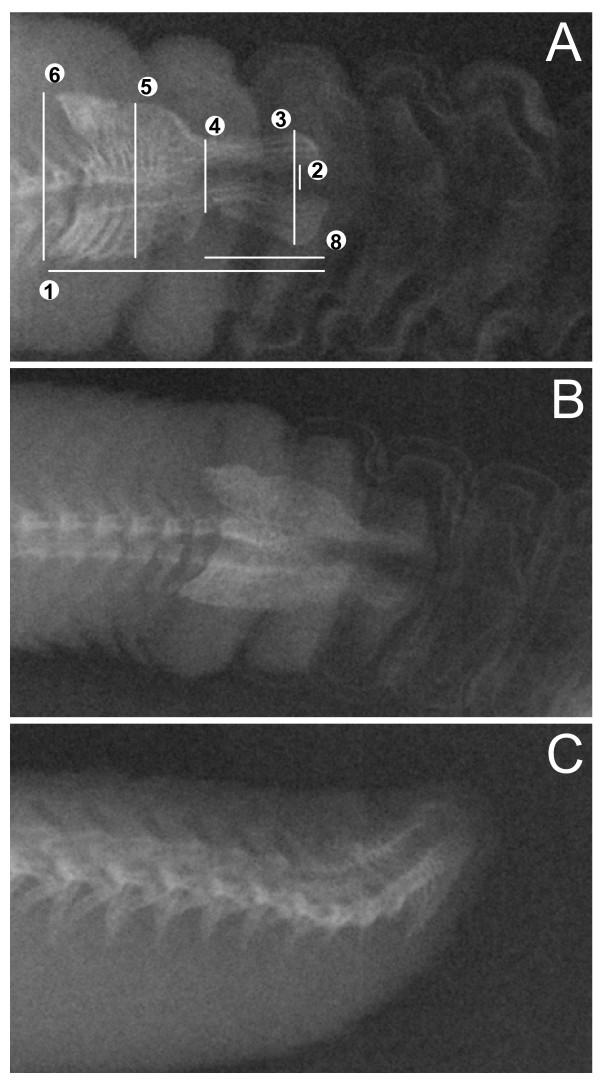
**Radiographs of representative rattlesnake styles**. (A) *Crotalus mitchellii *(UTA R-51444), showing linear measurements used in Evolutionary Principal Components Analysis (EPCA). Measurement 7 (not shown) is the transverse width of the style at its widest point. (B) *Crotalus ruber *(UTA R-7235). (C) *Crotalus catalinensis *(UTA R-32129).

We obtained digital radiographs from 34 species of rattlesnakes (1–9 specimens per species) using ethanol-preserved adult specimens (specimens examined are listed in Additional file 1, following abbreviations as in Leviton et al [15]). The vast majority of species from all major groups of rattlesnakes were sampled. Our sampling also included all three species of apparently rare long-tailed rattlesnakes from western Mexico (*Crotalus stejnegeri*, *C. lannomi*, and *C. ericsmithi*), which are collectively known from fewer than fifteen specimens. We imported radiographs into ImageJ 1.34s [16] and obtained eight linear measurements for each style (to nearest 0.01 mm). As homologous landmarks were ambiguous, we selected standardized measurements that were reproducible for most species (Fig. 1). A few species (e.g., *Sistrurus miliarius*, *C. stejnegeri*, etc.) did not exhibit certain typical style features (such as distal prongs, etc.); when such features were not present, we considered the measurement to be negligible (0.01 mm) for analytical purposes. In addition to style measurements, we collected data on snout-vent length (SVL), tail length, height and width of the exposed end-body lobe (i.e., basal rattle segment), and subcaudal scale counts. For quantitative analysis (see below), we included data only from specimens from which radiographs were obtained; however, we augmented our qualitative comparisons of style morphology by examining skeletal preparations. To clarify style structure in a basal rattlesnake, we also cleared and stained the tail of a large adult *S. miliarius *following the procedure in Cohn and Tickle [17]. The radiographs from this species did not show the typical arrowhead form of the style, but rather appeared to consist of few close-set, vertebral elements that were only slightly enlarged in the dorsal-ventral axis.

We examined interspecific variation in style morphology using Evolutionary Principal Components Analysis (EPCA). In cross-species comparisons, EPCA has an advantage over standard PCA in that EPCA extracts axes that maximize evolutionary change rather than ordinating static variation. In this variant of PCA, ancestral states are first reconstructed using squared-change parsimony and then eigenanalysis is performed on vectors of evolutionary change along each branch [18]. Thus, by incorporating phylogenetic information, EPCA is potentially able to reduce non-independence of cross-species data sets due to similarity through common descent. Prior to inclusion in EPCA, we log_10_-transformed all linear measurements and then regressed these variables against log_10_-transformed SVL and included the size-adjusted residuals in the analysis. We omitted SVL from the analysis and standardized all remaining variables (i.e., performed eigenanalysis on data extracted from the correlation matrix). Because tail length and subcaudal scale count are strongly influenced by sexual dimorphism we included only males in the EPCA (males were selected because they outnumber females in natural history collections, and thus we had more species represented by only males than by only females).

**Figure 2:**
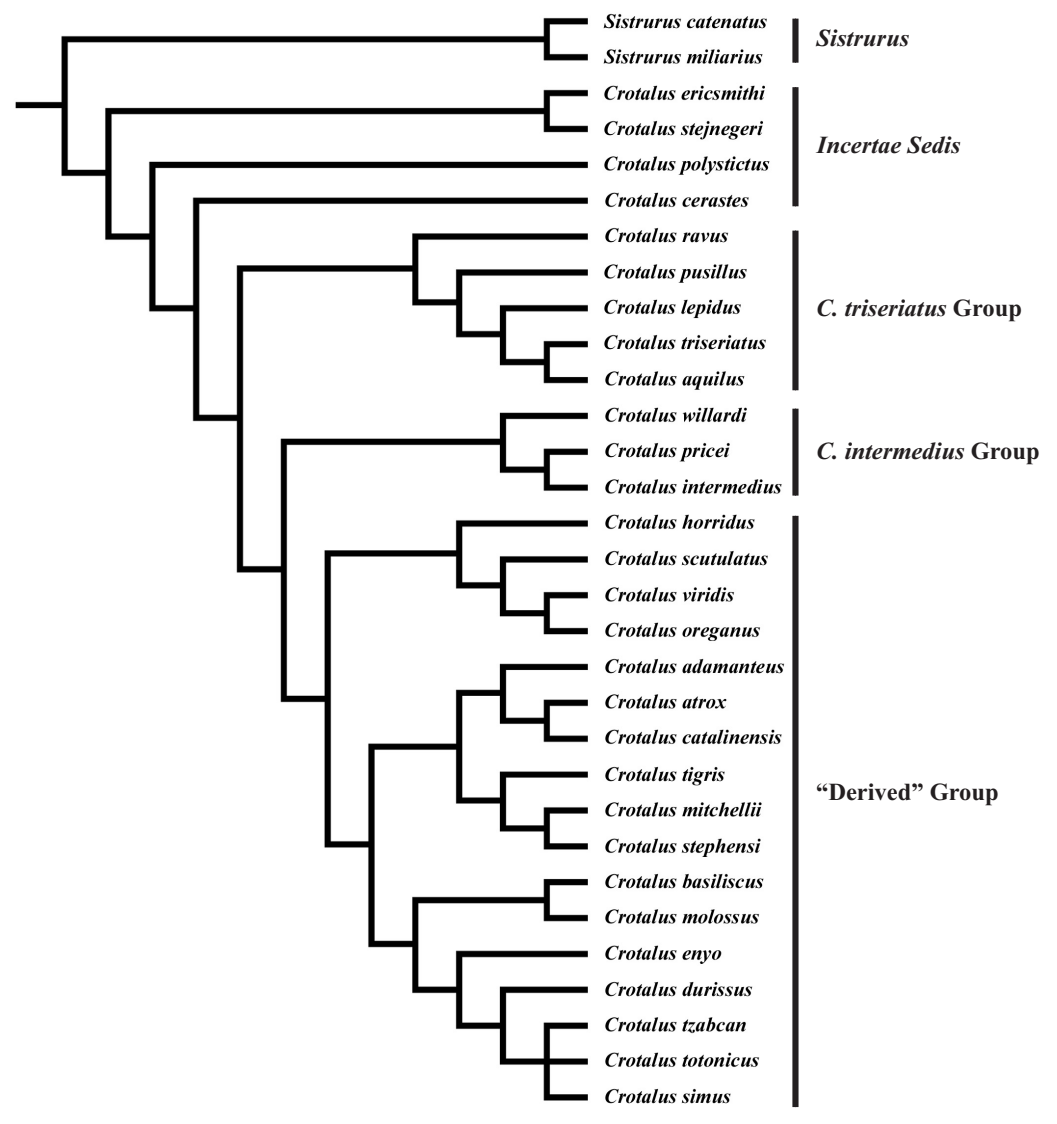
**Estimate of rattlesnake phylogeny used in this study**. The phylogeny is based on a composite of multiple sources (see text). "Derived" group includes species from four major clades (as delimited by Murphy et al. [19]).

We assembled a composite phylogeny for the taxa included in the EPCA using published studies of rattlesnake systematics [19-22] (Fig. 2), and set branch lengths equal to one. When nodal support was high (*Pp *> 0.95) we used the topology of Castoe and Parkinson [20] from their mixed model Bayesian analysis as the main basis for branching relationships; otherwise we used the preferred topology of Murphy et al. [19] as inferred using maximum parsimony. For our analysis we included the Mexican long-tailed rattlesnakes as the first clade diverging from within *Crotalus *[10,14,23-25], but recognize that these species, as well as *C. polystictus *and *C. cerastes*, remain *incertae sedis*. For purposes of visualization and discussion, we placed rattlesnakes into five groups based on our phylogenetic estimate (Fig. 2). We performed EPCA using the Rhetenor module [18] of Mesquite [26].

In addition to EPCA of radiograph measurements, we collated data on maximal ventral scale counts and maximal subcaudal scale counts (segmental counts) for males of all New World pitvipers, including rattlesnakes, to examine the association of dorsal (precloacal) and caudal vertebrae among species (data from [27]). There is a general 1:1 correlation between segmental scale counts and vertebral elements in snakes [28,29], and we confirmed this relationship for caudal vertebrae and subcaudal counts in rattlesnakes using radiograph data from selected individuals (excluding vertebrae coalesced into the style). Our objective was to examine the extent of separation (if any) between rattlesnakes and other species of New World pitvipers in the bivariate morphospace between dorsal and caudal vertebrae. Assuming that terminal vertebral elements in a generalized pitviper tail were reallocated to style formation after somitogenesis, providing a structural mechanism for the shortening of the rattlesnake tail and integration of the tail and rattle, the distribution of rattlesnake species in morphospace may inform whether certain aspects of style morphology preceded the coalescence of multiple vertebral elements into the style.

## Results

We interpreted the first two component axes extracted from the EPCA, which accounted for 75.5% and 10.4% of the total variation in the dataset, respectively. The first axis was structured primarily by the inverse relationship between style size and relative tail length/subcaudal scale count, with all eight style measurements loading positively on this axis (Table 1). This pattern likely reflects the general incorporation of additional caudal vertebral elements during the evolutionary increase in style size. The second EPCA axis reflected interspecific differences in style morphology, with style measurements pertaining to the distal third to half of the style contributing greater to variation in this axis (style measurements 5, 6, and 7 loaded negligibly on this axis). Style measurements 1, 2, and 8 were positively associated with EPC Axis 2, while style measurements 3 and 4 were negatively associated with this axis (Table 1). Thus we interpret the main spectrum of interspecific differences in style shape to reflect a continuum from relatively longer styles with deeper prongs, wider gaps between prongs (i.e., greater angle), more attenuated prongs, and greater constriction (or notching) near the juncture with the prongs and the anterior body of the style, to relatively shorter styles with shallow, but broad, prongs (or, in the extreme condition, prongs absent), and less constriction at the juncture of the prongs and the anterior style body (see Fig. 1).

**Table 1:** Character loadings and percentage of total variance explained for first two component axes extracted from Evolutionary Principal Components Analysis (EPCA)

Character	EPC 1	EPC 2
Relative tail length	-0.07	0.66
Subcaudal Scale Counts	-0.16	0.49
Basal rattle segment height	0.32	0.04
Basal rattle segment width	0.31	0.09
Style 1	0.34	0.30
Style 2	0.31	0.26
Style 3	0.29	-0.29
Style 4	0.31	-0.17
Style 5	0.32	-0.02
Style 6	0.33	-0.05
Style 7	0.29	0.01
Style 8	0.30	0.20
% of total variance explained	75.50	10.44

See Figure 1 for measurements taken from styles.

**Figure 3:**
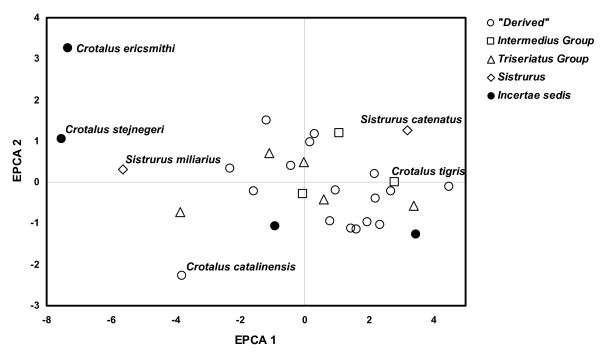
**Ordination of species factor scores for the first two axes from the Evolutionary Principal Components Analysis (EPCA)**. Markers indicate mean value for each species when more than one specimen was examined. See figure 2 for species content within each category.

**Figure 4:**
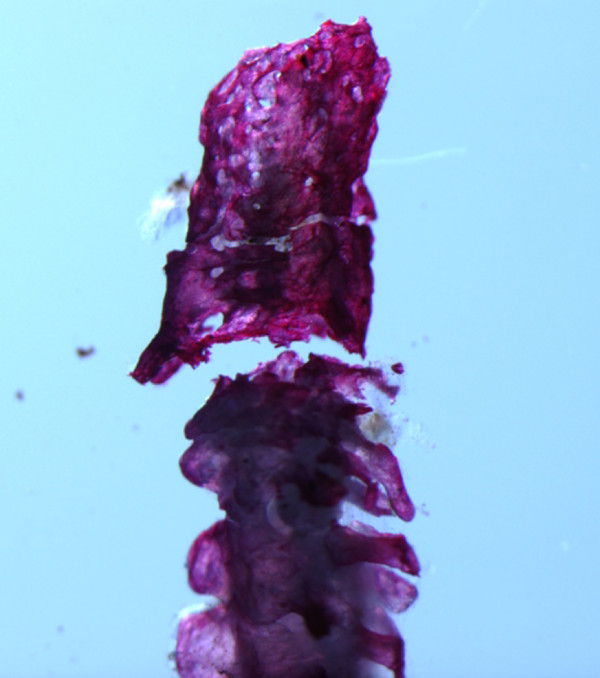
**Photograph of cleared and stained tail terminus of a large *Sistrurus miliarius*, showing aspects of style morphology and adjacent caudal vertebrae**.

A scatterplot of species factor scores showed that most species grouped near the plot origin, and that members of the *C. triseriatus *Group, *C. intermedius *Group, and "derived" species overlapped considerably in morphospace (Fig. 3). *Crotalus stejnegeri*, *C. ericsmithi*, and *Sistrurus miliarius *had exceptionally small styles whereas *C. tigris *had the relatively largest style – this species is notable in having a correspondingly large rattle. The most divergent styles in terms of general morphology were observed in *C. ericsmithi *and *C. catalinensis*. *Crotalus ericsmithi *possesses an exceptionally small style with two distinctive, albeit diminutive, prongs that project sharply ventrally and dorsally, respectively. *Crotalus catalinensis*, which does not usually retain rattle segments in life, has a blunt, triangular-shaped style without distinctive prongs or mid-style notching (Fig. 1). From radiographs, it appeared that *Sistrurus miliarius*, *C. lannomi*, and *C. stejnegeri *have similar tiny, amorphous styles. We further examined this apparent condition using a cleared and stained tail preparation of a large adult *S. miliarius *(Fig. 4). The preparation revealed the coalescence of relatively few (approximately 2–4) terminal vertebrae and the absence of prongs or distinct mid-style notching. The style of *S. miliarius *did exhibit similar bone porosity, texture, and structure to styles of more derived rattlesnake species as well as the degeneration of precise vertebral morphology (e.g., an absence of distinctive patterning or regularly repeated vertebral elements such as spines or processes). In contrast to its congener, *S. catenatus *has a relatively large, pronged style.

**Figure 5:**
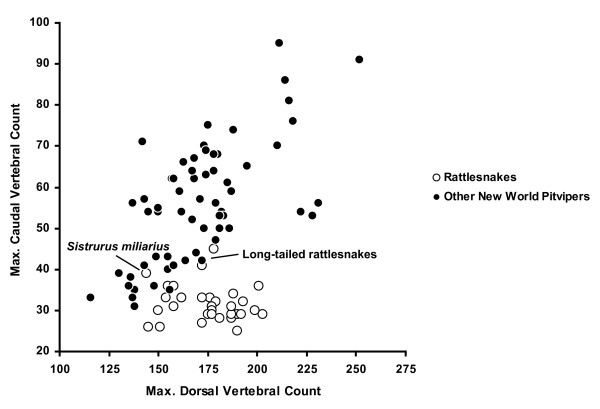
**Bivariate scatterplot of association between maximum precloacal and caudal segmental counts for rattlesnakes and other New World pitviper species**. With few exceptions (long-tailed rattlesnakes, *Sistrurus miliarius*) rattlesnakes show an apparent dissociation between segmental regions. Data presented are for males only to reduce the influence of sexual dimorphism.

There is a general evolutionary association between the number of precloacal and caudal vertebrae in snakes; however, there are various instances across the phylogenetic diversity of this group where this pattern is contradicted [30]. In New World pitvipers, there is a positive relationship between number of precloacal and caudal vertebrae; however, rattlesnakes are notably distinct in that with few exceptions they appear to possess relatively fewer caudal vertebrae than expected when compared to number of precloacal vertebrae (Fig. 5). Assuming that this dissociation is based on fusion of vertebrae and does not reflect an actual reduction in caudal vertebrae, the pattern further suggests the possibility that proportionately more vertebral elements are coalesced into the style in rattlesnake species with higher precloacal vertebral counts. This may be related to the observation that larger rattlesnake species tend to have correspondingly larger rattles. Three species (*Sistrurus miliarius*, *Crotalus ericsmithi*, and *C. stejnegeri*), which likely represent very early branching events within rattlesnakes, did not deviate from the generalized pitviper pattern of positive association between precloacal and caudal vertebrae counts.

## Discussion

Inferences regarding the evolution of the rattlesnake style necessarily require a robust hypothesis of rattlesnake relationships. Unfortunately, the rattlesnake phylogeny remains somewhat poorly resolved, despite initial analyses based on mitochondrial DNA sequence data [19,20]. Of particular interest are the three enigmatic long-tailed rattlesnake species from the Pacific versant of Mexico. These species are collectively known from fewer than fifteen specimens and have yet to be included in a formal phylogenetic analysis. The long-tailed rattlesnakes have generally been considered to represent an early divergence within *Crotalus *on the basis of putatively primitive character states, and they share additional color pattern and scalation features that may be found to be synapamorphic. Campbell and Flores-Villela [25] recognized that tail length and rattle size were not independent characters and further suggested that the proportionately long tail in these species may represent a plesiomorphic trait, and thus would be of no utility in resolving relationships based on parsimony. Although we agree with these authors regarding tail length in the general context of snake evolution, the phylogenetic position of rattlesnakes as nested within New World pitvipers, which are long-tailed when compared to rattlesnakes, suggests that the relatively shorter tail of rattlesnakes may be a secondarily derived condition.

Our study provides further insight into the question of whether the long-tailed condition is ancestral in rattlesnakes. Assuming subcaudal counts are a reasonable surrogate for tail length [29], the majority of rattlesnake species have considerably shorter tails than other New World pitvipers (Fig. 5). The process of tail shortening in rattlesnakes is likely a function of the relative contribution of caudal vertebral elements fused into the bony style (as opposed to evolutionary decrease in number of caudal segments); thus tail length is inversely related to rattle size. Since vertebral fusion comprises part of the rattle system, which is inarguably derived in rattlesnakes, the short-tailed condition is also derived in this group. However, we recognize that we cannot extend this argument to deciphering relationships within rattlesnakes, as reversals towards the longer-tailed condition are possible.

Combined evidence from EPCA and segmental regional association suggest a possible sequence of morphological transitions during style evolution. Largest magnitude differences in style morphology among rattlesnake species were in size-corrected measurements from the distal half of the style, which likely correspond to aspects of matrix morphology. Size-corrected measurements from the proximal half of the style (including style width in the transverse plane) were conserved across species, perhaps reflecting a general lack of evolutionary variation in tailshaker muscle attachment sites. The style of *Sistrurus miliarius*, a basal rattlesnake species, is notable in that it comprises very few caudal vertebral elements, and lacks mid-style notching and distal prongs (Fig. 4). The style does show foramina and minimal proximal flaring; but otherwise consists of vertebrae that lack precise patterning in the form of processes and spines. This reduced style morphology is shared with *C. stejengeri *and *C. lannomi*. *Crotalus ericsmithi *is similar in that very few vertebral elements comprise the style; however, this species is distinctive in having tiny distal prongs that form a broad angle. The substantial overlap around axis means in ordination space of the *C. triseriatus *Group, *C. intermedius *Group, *C. cerastes*, *C. polystictus*, and the derived species implies that the derived morphology of large, globose, pronged styles comprised of multiple caudal vertebrae evolved relatively early in the rattlesnake radiation, and we propose that variation around this mean represents evolutionary refinements of the optimized style morphology. The most parsimonious scenario based on our phylogenetic placement of the Mexican long-tailed rattlesnakes as the earliest divergence within *Crotalus*, and the positive association of precloacal and caudal vertebrae in the nearest pitviper outgroups, suggests parallel evolution of the derived style morphology in *S. catenatus*, which also possesses a correspondingly large rattle when compared to *S. miliarius*.

Intriguingly, in the evolutionary history of rattlesnakes there appears to have been a contemporaneous apparent dissociation in the number of precloacal and caudal vertebrae (Fig. 5), which we interpret as an increase in the number of vertebral elements incorporated into the style, and an optimized refinement in style morphology towards the morphospace cloud occupied by the vast majority of extant species (Fig. 3). Assuming our phylogenetic estimate is correct regarding the placement of the Mexican long-tailed rattlesnakes, this event happened subsequent to both the divergence of *Sistrurus *and the basal split within *Crotalus*. Based on the pattern of species ordination, we suggest that the ability to retain functional rattle segments is correlated with both the size and morphology of the style (and by extension the rattle matrix). *Crotalus ericsmithi*, which presumably diverged along with the remaining long-tailed rattlesnakes before the rapid increase in vertebral element incorporation into the style, has an exceptionally diminutive rattle string. We hypothesize that the divergent style morphology in this species represents an alternative evolutionary path towards rattle segment retention that led to the adjustment of morphology of the prongs rather than an increase in rattle size. Within the derived rattlesnake group, *Crotalus catalinensis *has the most divergent style. This is notable in that *C. catalinensis *is also the only species examined that has evolutionarily lost the retention of interlocking rattle segments. The style of this species is reduced and blunt, and does not have distinctive distal prongs (Fig. 1). In comparisons of various populations of insular rattleless populations and their mainland derivatives, Rabatsky [13] found consistent differences in the morphology of the end-body matrix associated with the loss of rattle strings. Our results support the idea that morphological vestigilization extends to the bony style as well.

Although it is evident that style morphology is associated with rattle, and more specifically rattle matrix morphology, we are currently unable to distinguish between potential scenarios regarding the evolutionary integration of these two components. It is possible that selection acts disproportionately on matrix morphology to retain rattle segments, and that style morphology evolves through pleiotropic effects of gene regulatory programs involved in the secretion of keratin, patterning, or growth and resorption of the surrounding matrix tissues. In contrast, the style may have been the target of stronger selective forces as the style provides an anatomical link between the rattle-forming matrix tissues and the tailshaker muscles that drive the high-frequency oscillations of the rattle. Alternatively, style and matrix may evolve independently under differing selective regimes.

Without additional data we favor the hypothesis that matrix morphology (as opposed to style morphology) is a direct target of selection for the following reasons: (1) although independent evolution and subsequent integration of components is possible, it is less parsimonious than assuming that anatomical elements of the rattle system evolved in an incremental modular fashion; (2) rapid tail vibration is a plesiomorphic trait in rattlesnakes [7], thus derived tailshaker muscles likely represent a refinement of the rattle system not a prerequisite, negating the necessary link between tailshaker musculature and the rattle itself; (3) matrix morphology is both more precise than style morphology and more directly influences rattle segment form; and (4) the rattle is the phenotypic component of the system that most directly interfaces with the putative selective force (i.e., defense against predation). Further phylogenetic analysis of other components of the rattle system, and the analysis of gene expression patterns and interactions between the preemptive rattle system components may partially test the hypothesis presented here that style morphology evolves indirectly as an integrated module with the developmental patterning of the keratin-secreting matrix tissues.

## Conclusion

The evolution of the rattlesnake style is characterized by two independent transitions from small styles composed of few coalesced vertebral elements to large, globose styles composed of many caudal vertebrae. General morphological correlates of style and rattle morphology suggest that size and shape of the style are related to rattle morphology and the retention of rattle segments; however, as the rattle is an integrated system, we do not suggest that selection on style morphology predominately drives evolution of rattle morphology. Many authors have speculated on the evolutionary context of the origin of the rattle system [e.g., [7,31,32]], but as phylogenetic relationships become resolved a more rigorous comparative approach to this question can be attained by examining the sequence of evolution of the various rattle system components, both individually and in concert.

## Competing interests

The authors declare that they have no competing interests.

## Authors' contributions

JMM analyzed style data and drafted the initial manuscript. Both JMM and APD conceived the study, collected data, interpreted analyses, and finalized the manuscript.

## Supplementary Material

Additional file 1 **Specimens examined.** Collection abbreviations are from Leviton et al. [15]. Click here for file
