# Supplementary material for: Evolutionary morphology of the rattlesnake style
Source: BMC Evol Biol. 2009 Feb 10;9:35. doi: 10.1186/1471-2148-9-35 (PMC2645363; doi:10.1186/1471-2148-9-35)
Supplement: Additional file 1 — Specimens examined. Collection abbreviations are from Leviton et al. [15]. [file 1471-2148-9-35-S1.doc]

Additional File 1 – Specimens Examined

*Agkistrodon contortrix*: UTA R-32200. *Crotalus adamanteus*: UTA R-11372. *C. aquilus*: UTA R-12594, 12967. *C. atrox*: UTA R-5583, 16222, 17121, 18528–29, 22355, 27200, 32188, 46023, 54367–68, 55393–94. *C. basiliscus*: UTA R-6120. *C. catalinensis*: UTA R-32129. *C. cerastes*: UTA R-45170, 45928. *C. durissus*: UTA R-35936. *C. enyo*: UTA R-7617, 8082. *C. ericsmithi*: UTA R-55372. *C. horridus*: UTA R-26533–34. *C. intermedius*: UTA R-4539, 51457. *C. lannomi*: BYU 23800. *C. lepidus*: UTA R-7434, 34539. *C. mitchellii*: UTA R-51431, 51444. *C. molossus*: UTA R-34139, 38680. *C. oreganus*: UTA R-17305. *C. polystictus*: UTA R-5667. *C. pricei*: UTA R-9661, 16289. *C. pusillus*: UTA R-5846, 9358. *C. ravus*: UTA R-32620. *C. ruber*: UTA R-7235. *C. scutulatus* UTA R-44376. *C. stejnegeri*: LACM 37718; UTA R-6234. *C. stephensi*: UTA R-51442. *C. tancitarensis*: UTA R-52401. *C. tigris*: UTA R-31060, UTA uncatalogued specimen. *C. totonicus*: UTA R-7222. *C. triseriatus*: UTA R-12600. *C. tzabcan*: UTA R-52497. *C. unicolor*: UTA R-8199. *C. viridis*: UTA R-40914. *C. willardi*: UTA R- 22526, 32081. *Sistrurus catenatus*: UTA R-22372, 53740. *S. miliarius*: UTA R-1288, 18029, 18364. 26539–40, 35216–18, 44588, 55411.
